# Supplementary material for: Toxicity of Asciminib in Real Clinical Practice: Analysis of Side Effects and Cross-Toxicity with Tyrosine Kinase Inhibitors
Source: Cancers (Basel). 2023 Feb 7;15(4):1045. doi: 10.3390/cancers15041045 (PMC9954054; doi:10.3390/cancers15041045)
Supplement: Supplementary file 1 [file cancers-15-01045-s001.zip › cancers-2195682-supplementary.pdf]

Supplementary Material

	Frequencies of AEs			Cross-Intolerance analysis			
				Non-PTT(n=51), %		PTT (n=26), %	
	Total (n=77), n (%)	Non-PPT (n=51), n (%)	PTT (n=26), n (%)	Frequency in patients with that previous AE	Frequency in patients without that previous AE	Frequency in patients with that previous AE	Frequency in patients without that previous AE
Thrombocytopenia	13 (16.9)	8 (15.7)	5 (19.2)	53.3%	0%	30.8%	7.7%
Anemia	9 (11.7)	6 (11.8)	3 (11.5)	25%	3.2%	16.7%	7.1%
Neutropenia	5 (6.5)	2 (3.9)	3 (11,5)	11.1%	2,4%	30%	0%
Fatigue	14 (18.2)	10 (19.6)	4 (15.3)	33.3%	15.4%	37.5%	5.5%
Arthralgias	9 (11.7)	7 (13.7)	2 (7.7)	28.6%	8.1%	12.5%	5.5%
Nausea	6 (7.8)	4 (7.8)	2 (7.7)	15.4%	5.2%	11.1%	5.9%
Pleural/pericardial effusion	4 (5.2)	3 (5.9)	1 (3.8)	10%	3.2%	11.1%	0%
Pancreatitis	2 (2.6)	1 (1.9)	1 (3.8)	33%	0%	33%	0%

Table S1. Adverse effects and cross-intolerance comparison between ponatinib pretreated patients and non-ponatinib pretreated patients.
AE: adverse effects. PTT: Ponatinib pretreated patients.
